# Microstructure and dielectric properties of biocarbon nanofiber composites

**DOI:** 10.1186/1556-276X-8-293

**Published:** 2013-06-22

**Authors:** Bo Dai, Yong Ren, Gaihua Wang, Yongjun Ma, Pei Zhu, Shirong Li

**Affiliations:** 1 State Key Laboratory Cultivation Base for Nonmetal Composites and Functional Materials, School of Materials Science and Engineering, Southwest University of Science and Technology, Mianyang 621010, People’s Republic of China; 2 Analytical and Testing Center, Southwest University of Science and Technology, Mianyang 621010, People’s Republic of China

**Keywords:** Carbon fibers, Heat treatment, Electrical properties, Transmission electron microscopy (TEM)

## Abstract

A kind of web-like carbon with interconnected nanoribbons was fabricated using bacterial cellulose pyrolyzed at various temperatures, and the microwave dielectric properties were investigated. Bacterial cellulose was converted into carbonized bacterial cellulose (CBC) with a novel three-dimensional web built of entangled and interconnected cellulose ribbons when the carbonization temperature was below 1,200°C; the web-like structure was destroyed at a temperature of 1,400°C. Composites of CBC impregnated with paraffin wax exhibited high complex permittivity over a frequency range of 2 to 18 GHz, depending on the carbonization temperature. Both real and imaginary parts were the highest for CBC pyrolyzed at 1,200°C. The complex permittivity also strongly depended on CBC loadings. For 7.5 wt.% loading, the real and imaginary permittivities were about 12 and 4.3, respectively, and the minimum reflection loss was -39 dB at 10.9 GHz. For 30 wt.% loading, the real and imaginary permittivities were about 45 and 80, respectively, and the shielding efficiency was more than 24 dB in the measured frequency range and could be up to 39 dB at 18 GHz. The electromagnetic properties were assumed to correlate with both the dielectric relaxation and the novel web-like structure.

## Background

The rapid proliferation of advanced electronic devices for many commercial and military applications, such as data transmission, telecommunications, wireless network systems, and satellite broadcasting as well as radar and diagnostic and detection systems, has led to numerous electromagnetic compatibility and electromagnetic interference (EMI) problems. The interaction of electromagnetic waves originating from different sources can lead to a decrease in quality and a misinterpretation of transferred data, and it has thus become vital to avoid such interference and electromagnetic wave pollution through the use of appropriate absorbing and shielding materials. Carbonaceous materials - such as graphite and/or carbon black - are often used as dielectric electromagnetic absorbers, generating dielectric loss by improving the electrical conductivity of the mixture. In particular, nanostructured materials and carbon fiber composites have been the subjects of growing interest as microwave radiation absorbing and shielding materials in the high-frequency range due to their fascinating properties [1-5]. It is reported that carbon nanofiber-polymer composites presented EMI shielding effectiveness (SE) of approximately 19 dB with 15 wt.% carbon nanofiber loading [3]. Graphite-coated FeNi nanoparticles exhibited reflection loss (RL) of approximately -23 dB with the thickness 2.5 mm and the absorption peak at 14 GHz [5]. Carbon nanocoils coated with Fe_3_O_4_ exhibited remarkably improved microwave absorption (RL approximately -20 dB) compared to the pristine carbon nanocoils (RL approximately -2 dB) [6]. Another allotrope of carbon, viz., single-layered two-dimensional graphene, graphene oxide, or reduced graphene oxide, has attracted a great deal of attention for its application in many diverse areas due to its unique electrical, mechanical, and thermal properties in addition to its light weight, high surface area, and layered morphology. The graphene/epoxy composites exhibited SE of approximately 21 dB in the *X*-band for a 15 wt.% loading [7]. The reduced graphene oxide exhibits -7 dB RL while graphite only exhibits approximately -1 dB in the frequency range of 2 approximately 18 GHz [8]. Further to the considerable interest in adding small concentrations of nanocarbons into the matrix, what unquestionably matters is the ability to disperse them [9]. The cost and limited supply also hinders the application of nanocarbons as fillers for EMI shielding and microwave absorption. Recently, researchers have tried low-cost natural materials (rice husks) as carbonaceous sources to fabricate carbon-matrix composites with self-assembly interconnected carbon nanoribbon networks [10]. These composites have higher electric conductivities and EMI shielding effectiveness values than those without. In this paper, the example of microwave composites is reported using bacterial cellulose as the carbonaceous source, which had self-assembled interconnected nanoribbon networks. These composites exhibited high permittivity in the frequency range of 2 to 18 GHz and thus could be excellent high-loss materials, for example, as an EMI material or high-performance microwave absorbing material. The interesting electromagnetic characteristics are due to the novel three-dimensional web-like networks which establish additional electrical conduction pathways throughout the whole system.

## Methods

### Sample preparation

Carbonized bacterial cellulose (CBC) was obtained by heat-treated bacterial cellulose (BC), which was pyrolyzed for 4 h under a nitrogen atmosphere at 800°C, 1,000°C, 1,200°C, or 1,400°C. CBC was cleaned using diluted hydrochloric acid with volume fraction of 10% and then soaked in concentrated nitric acid at room temperature for 4 h. Afterwards, the black solution was diluted with distilled water and rinsed for several times until the pH value reaches 7. The resulting CBC were separated from the solution by filtration and dried using a vacuum at 60°C for further use. Dried CBC fibers were mechanically milled into powder for the measurement of electromagnetic parameters. The CBC/paraffin wax samples were prepared by uniformly mixing the powders in a paraffin wax matrix. A series of CBC/paraffin wax composites were prepared with CBC loading of up to 30 wt.%. The absorbers were dispersed in ethanol with paraffin wax by stirring and sonication at 90°C for 1 h. The mixtures were then pressed into cylindrical dies with 7.0 mm outer diameter, 3.0 mm inner diameter, and about 2.0 mm height.

### Characterization

The morphology of CBC was observed by transmission electron microscopy (TEM, Tecnai F20, FEI, Hillsboro, OR, USA) and scanning electron microscopy (SEM, FEI NOVA600i). The sheet resistance (*R*_s_) of the composites was measured by the four-probe method using a Keithley 2400 multimeter (Cleveland, OH, USA), and the direct current (DC) conductivity *σ* was obtained using the measured *R*_s_ and the sheet thickness *t* according to *σ* = 1/(*R*_s_*t*). Complex permittivity and permeability measurements were performed on an Agilent E8363B vector network analyzer in the 2 to 18 GHz frequency range. Three samples were tested for each electromagnetic parameter measurement, and the reported results are the averages.

## Results and discussion

### Phase and microstructure of CBC

**Figure 1:**
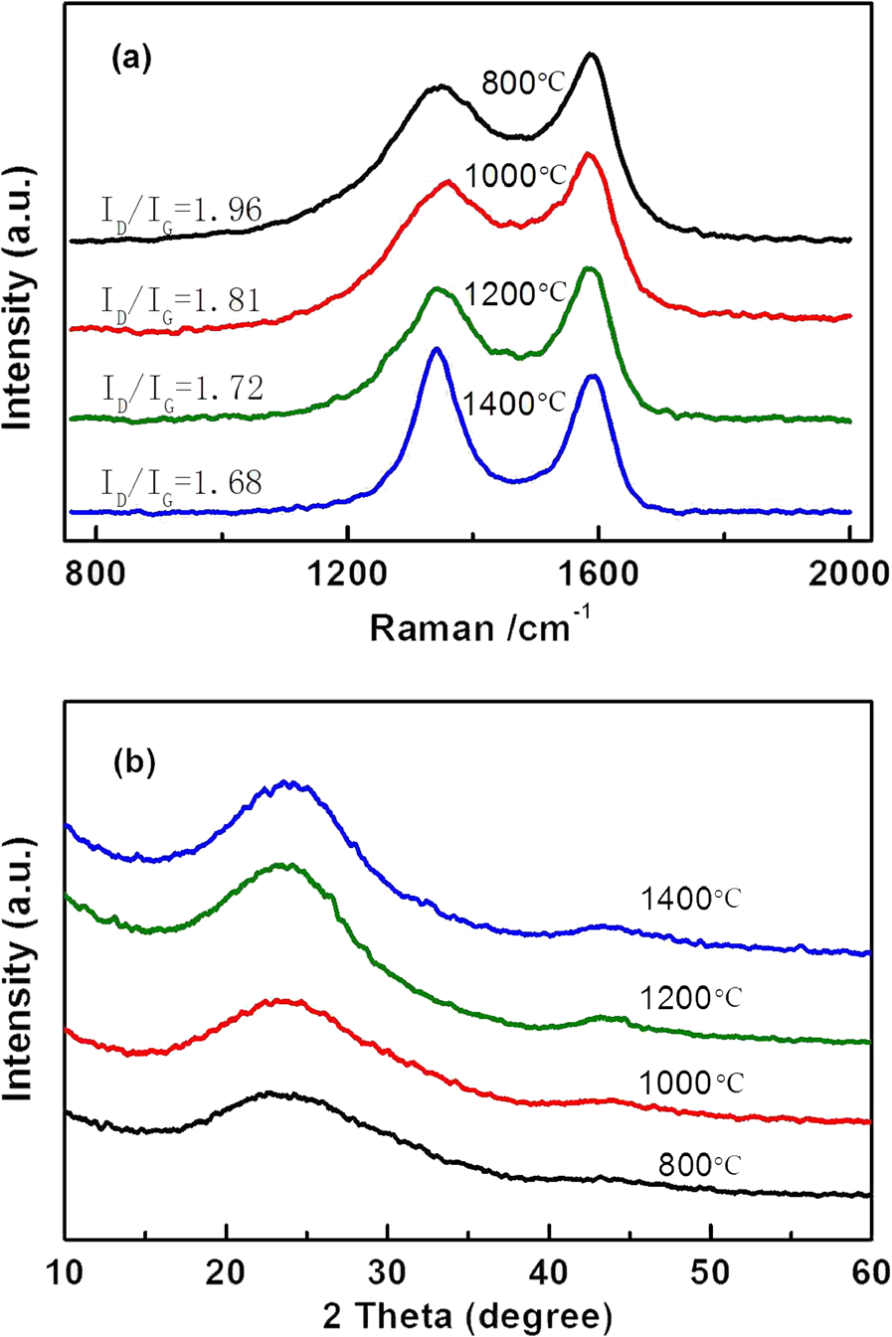
Raman spectra (a) and XRD patterns (b) for CBC pyrolyzed at various temperatures.

Raman scattering is a well-accepted characterization method for evaluating the degree of structural order of carbonaceous materials, using the ratio of the integrated intensity of the D band (*I*_D_) to that of the G band (*I*_G_) [11]. The typical Raman spectra (in a shift regime) of the CBC samples treated at various temperatures are shown in Figure 1a. It displays a prominent G-peak at approximately 1,585 cm^-1^ along with a D-peak at approximately 1,340 cm^-1^ corresponding to the first order scattering of the E_2g_ mode and A_1g_ mode, respectively. There are changes in the ratio of the area for the peaks assigned to the D and G bands, i.e., from 1.96 at 800°C to 1.68 at 1,400°C. The decrease in the ratio of the D/G bands may be explained in terms of an increase in the crystallite domains or a reduction in the quantity of amorphous carbon. Figure 1b shows the X-ray diffraction patterns of samples. It presents diffraction patterns typical of a predominantly amorphous carbon. The increased temperature led to an increase in their crystallinity, which corresponds to the result of Raman measurements.

**Figure 2:**
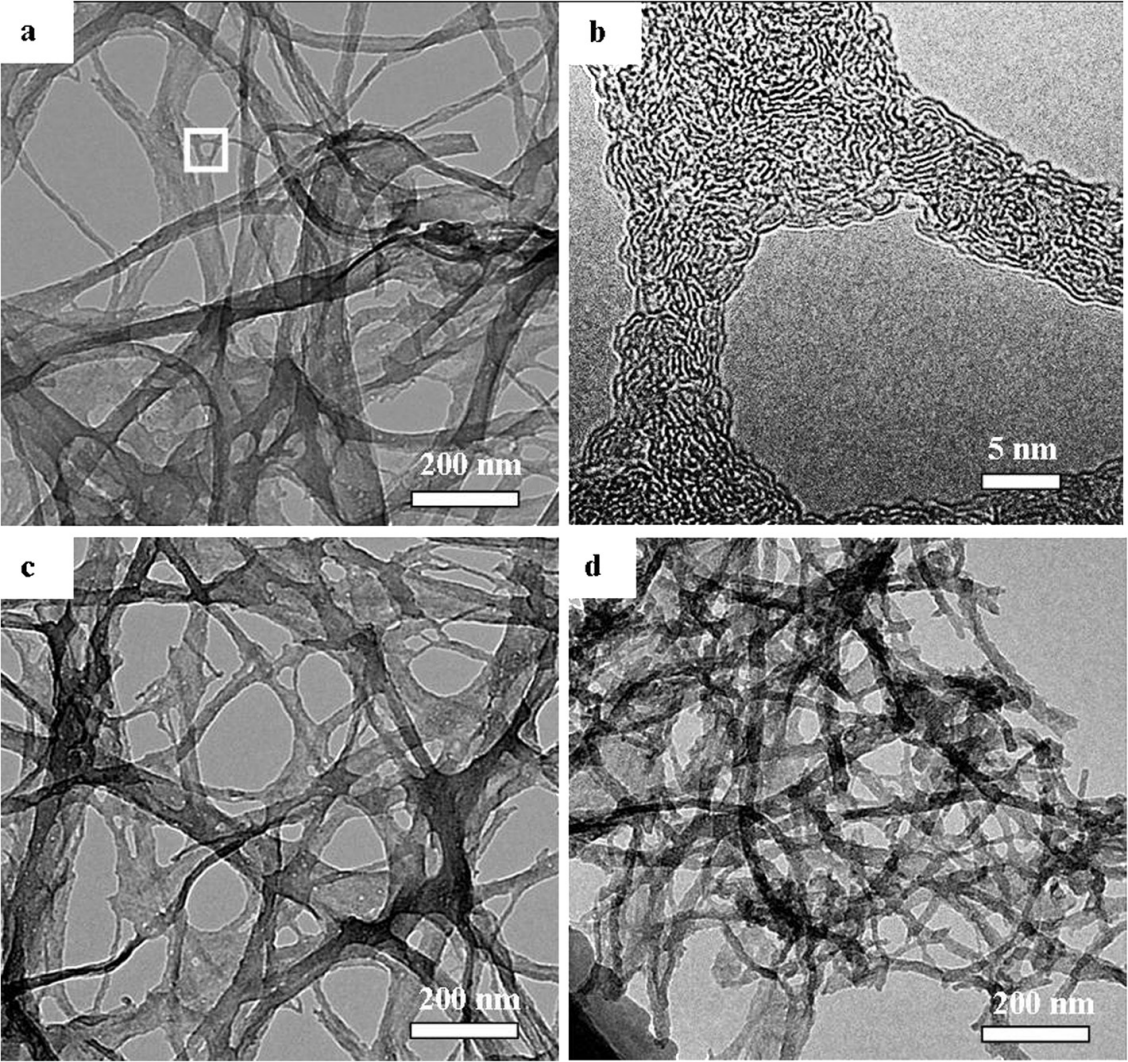
**TEM images of CBC pyrolyzed.** At (**a,b**) 800°C, (**c**) 1,200°C, and (**d**) 1,400°C, respectively.

BC fiber is an extracellular product excreted in the form of pellicles. It is structured in a web-like network by self-assembly of continuous nanofibers about 10 nm thick and 50 nm wide [12]. Each nanofiber is a bundle of cellulose microfibrils, each of which is about 4 nm thick and 4 nm wide. The web-like network leads BC to be homogenously dispersed in the matrices [13], and its composites have significant mechanical strength and extremely low thermal-expansion coefficients [14,15]. After carbonization under a nitrogen atmosphere, BC was converted into a kind of carbon nanoribbon and the corresponding TEM images are presented in Figure 2. As shown in Figure 2a, the carbonization at temperature of 800°C did not break the pristine structure, and the web-like networks were very well preserved. The carbonized bacterial cellulose networks can be described as a three-dimensional web built of entangled and interconnected cellulose ribbons. The width and thickness of the nanoribbons are in the order of tens of nanometers and a few nanometers, respectively. A higher magnification shows that each ribbon assembly is composed of a number of extended chains of bacterial fibrils (Figure 2b). These fibrils are seen to be in close contact with one another and to twist as a whole. The structure of BC carbonization at 1,200°C is almost the same as that of carbonization at 800°C, which formed branched nanoribbon networks. However, after carbonization at 1,400°C, branches of the nanoribbon seemed to be broken and the three-dimensional structure degraded to two dimensions. The width of the nanoribbon was narrower than those shown in Figure 2a,c.

### Microwave electromagnetic properties of CBC

**Figure 3:**
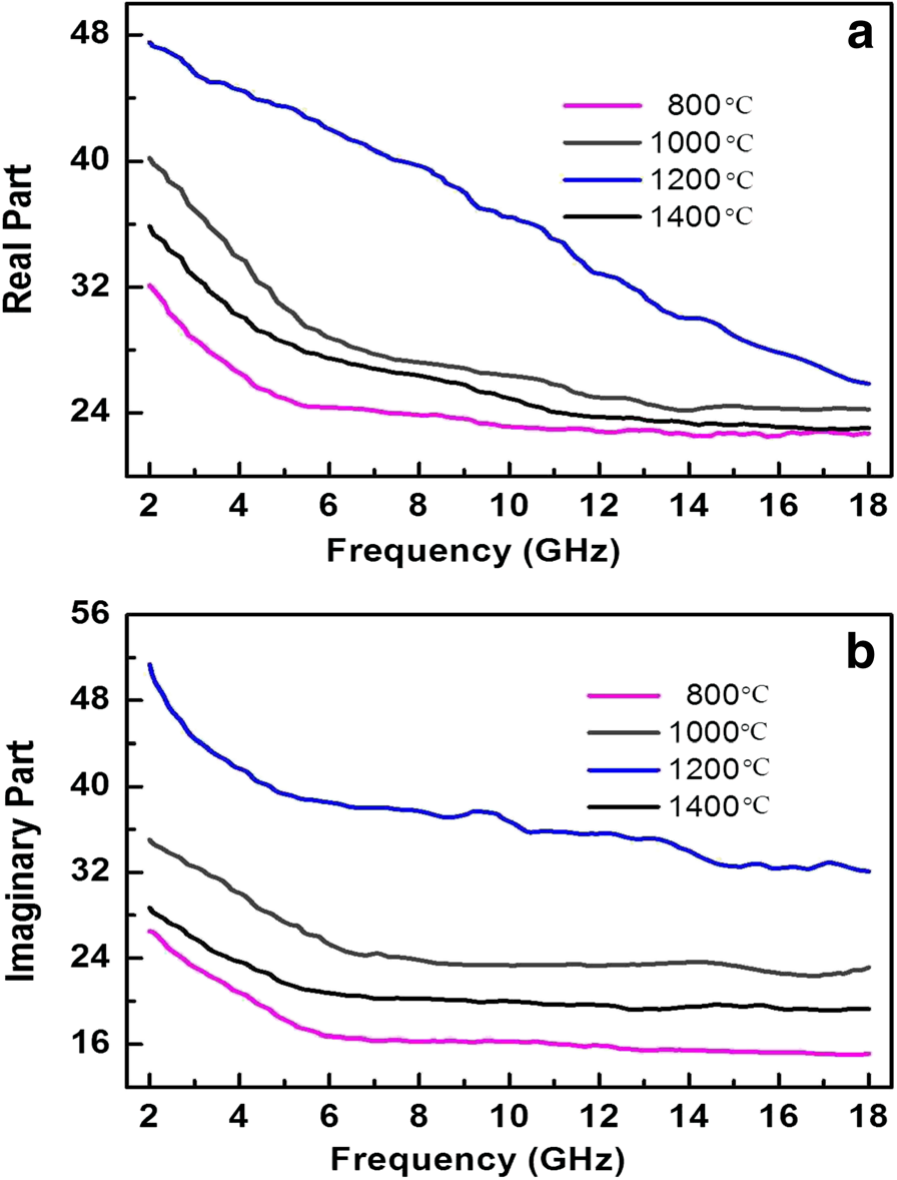
Real (a) and imaginary (b) parts of permittivity for the composites with 20 wt.% CBC loadings.

The relative complex permittivity (*ϵ*_*r*_ *= ϵ′ - jϵ″*) was measured in the frequency range of 2 to 18 GHz. The real (*ϵ′*) and imaginary (*ϵ″*) parts of permittivity for the composites with 20 wt.% CBC loadings pyrolyzed at different temperatures are presented as a function of the frequency in Figure 3a,b. Both the real and the imaginary permittivities presented high values. The complex permittivity spectra reveal the behaviors of electrical conduction and dielectric relaxation of the composites. Upon increasing the temperature, the permittivity plots for the specimen displayed a firstly increasing and then diminishing response*.* At 1,200°C, the values of both *ϵ′* and *ϵ″* were the highest. The two mechanisms responsible for the dielectric properties were analyzed. First, there are many mobile charge carriers (electrons or holes) with great mobility in CBC that interact with electromagnetic fields by oscillating when irradiated, just like those in carbon nanotubes (CNTs). Second, it is proposed that the web-like networks in CBC also established bridges for mobile charge carriers along which they can move freely. These additional channels interact with the electromagnetic field over a short range, resulting in high permittivity. With an increase in the pyrolysis temperature, the degree of graphite order increased as discussed above; and thus, there were more mobile charge carriers. However, the web-like networks of carbon nanofiber was somehow destroyed when the pyrolysis temperature increased beyond 1,200°C (as shown in Figure 2d). Therefore, it is understandable that the CBC pyrolyzed at 1,200°C exhibited the highest permittivity. In addition, it is noteworthy that the magnitudes of the loss tangent (tan *δ*_*e*_ = *ϵ*″/*ϵ*′) approached 1, even exceeded 1, especially for that sample pyrolyzed at 1,200°C. The loss tangent is directly related to the attenuation factor *α* that determines the attenuating property of the material: the higher the loss tangent, the larger the attenuation factor [16]. Therefore, the high loss tangent for the CBC composites signifies that they have good attenuating properties.

**Figure 4:**
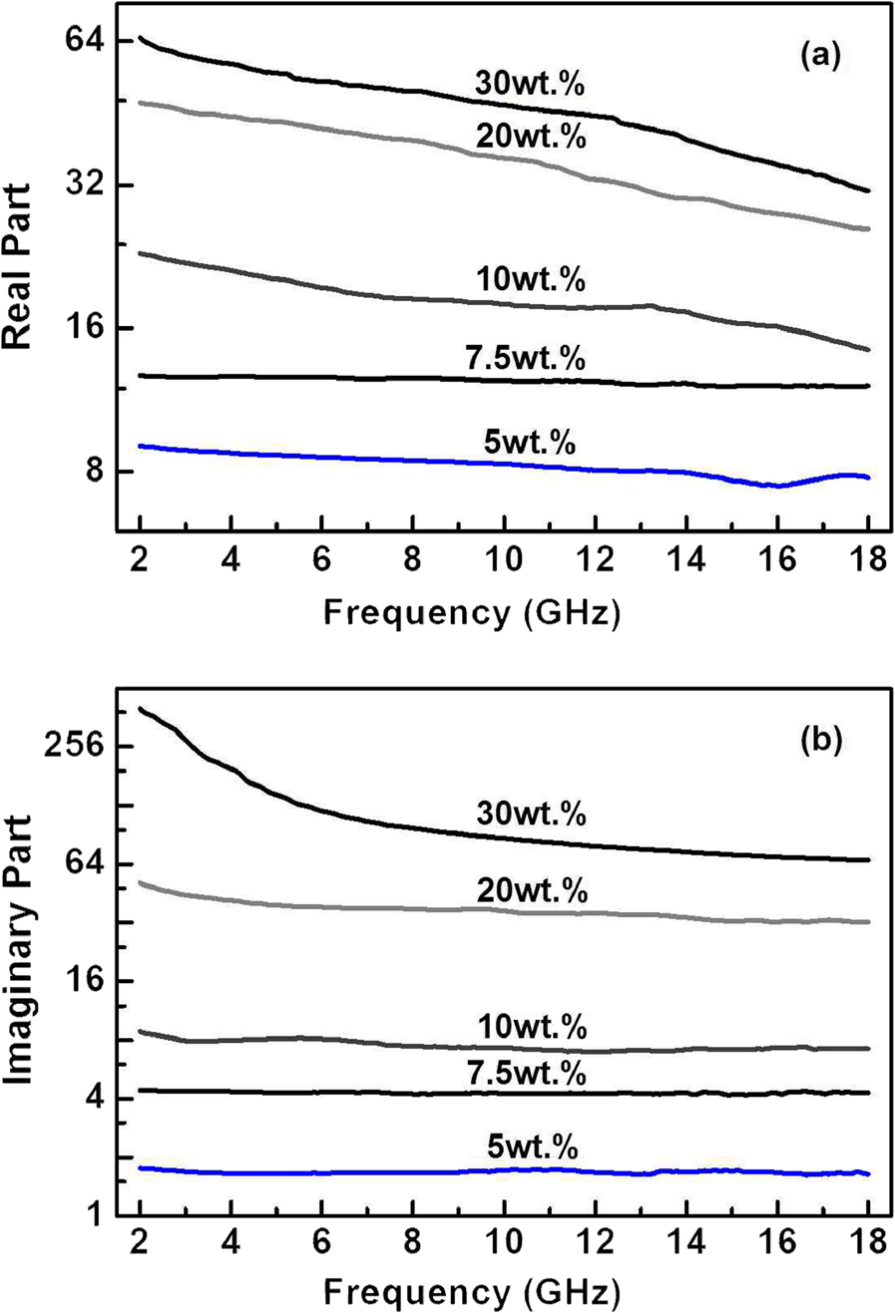
Frequency dependencies of (a) real and (b) imaginary permittivities.

**Figure 5:**
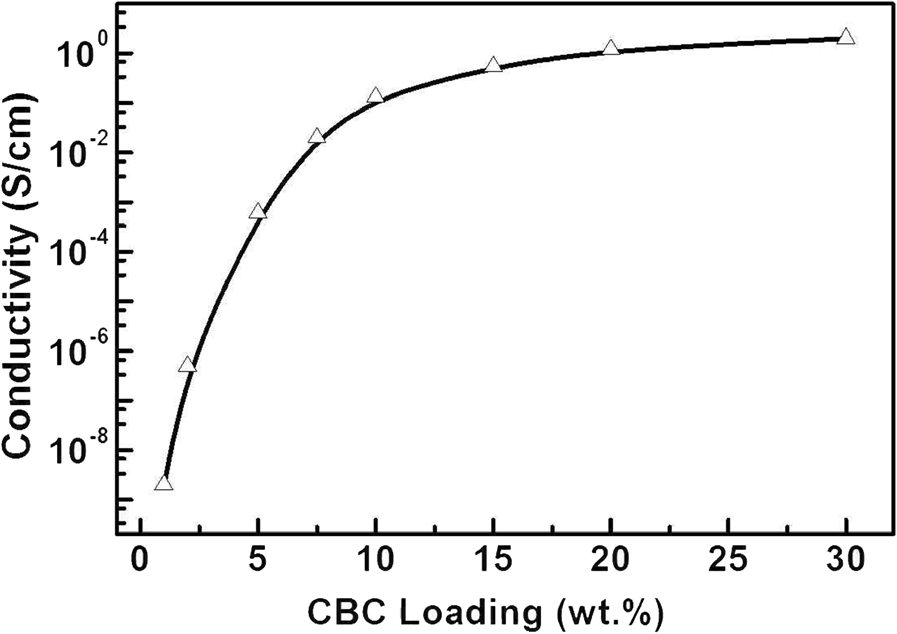
DC conductivity of CBC/paraffin wax composites versus CBC loading pyrolyzed at 1,200°C.

Figure 4 shows the dielectric permittivities of the CBC paraffin wax composites with 5 to 30 wt.% CBC pyrolyzed at 1,200°C. It is evident that both the real and imaginary permittivities increased rapidly with CBC concentration. The complex permittivity spectra reveal the behavior of electrical conduction and dielectric relaxation of the composites. The rapid increase in the permittivities with concentration is attributed to the onset of percolation, similar to that of the CNTs [17,18]. Figure 5 is a plot of DC conductivity of the CBC/paraffin wax composites versus the amount of the CBC loading pyrolyzed at 1200°C. One can see a sharp increase of conductivity when CBC loading was increased from 1 to 7.5 wt.%. The conductivity of the CBC was of 2 × 10^-9^ S/cm for 1 wt.% and 0.02 S/cm for 7.5 wt.% and reached a relatively high value of 0.5 S/cm for 15 wt.%. This implies that such a composite has a percolation threshold of about 7.5 wt.%.

**Figure 6:**
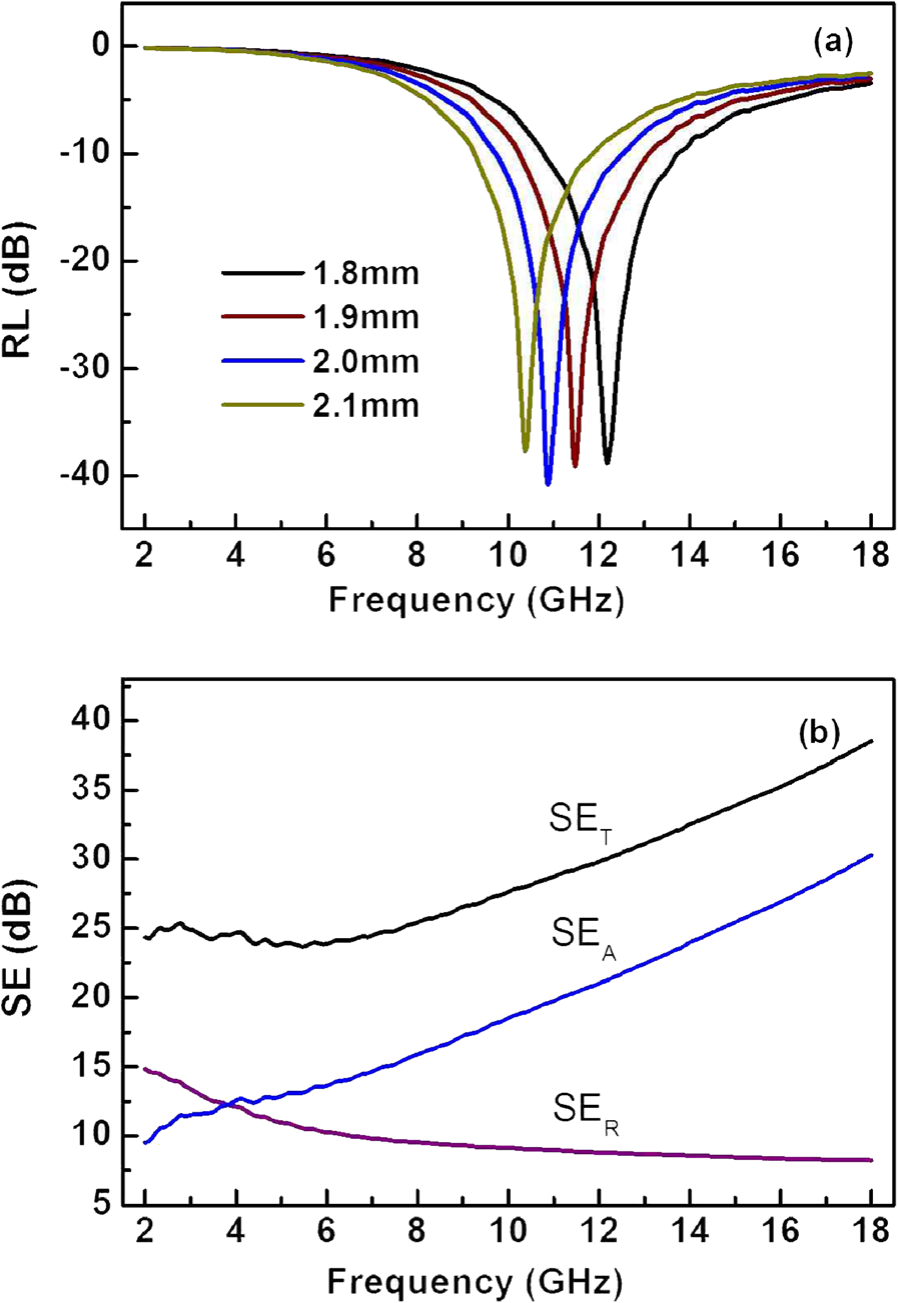
Frequency dependences of the RL at various sample thickness (a) and the EMI shielding efficiency (b).

For microwave absorption, the elelctromagnetic parameters should be appropriate, and the optimal filler concentration is always around the percolation threshold. Theoretical RL values in the sample with 7.5 wt.% CBC loading were calculated according to the transmission line theory [19].

(1) $$\mathrm{RL}=20\mathrm{lg}\left|\left(Z_{\mathrm{in}}-1\right)/\left(Z_{\mathrm{in}}+1\right)\right|$$

(2) $$Z_{\mathrm{in}}=\sqrt{\mu_{r}/\epsilon_{r}}\text{tanh}\left[j\left(2\mathrm{\pi ft}/c\right)\sqrt{\mu_{r}\epsilon_{r}}\right]$$

where *Z*_in_ is the normalized impedance at the absorber surface. Figure 6a shows the frequency dependences of the RL at various sample thickness (*t* = 1.8, 1.9, 2.0, and 2.1 mm). An optimal RL of -40.9 dB was observed at 10.9 GHz with the -20 dB bandwidth over the frequency range of 10.4 to 11.4 GHz for *t* = 2.0 mm. The minimum RL obviously shifts to lower frequency range with increased thickness, which can be understood according to the geometrical effect matching condition in which the thickness of the layer is a quarter wavelength thickness of the material. It is interesting that microwave absorption properties do not change dramatically for the thicknesses of 1.8 to 2.1mm.

For EMI shielding, the total shielding effectiveness SE_*T*_ is always expressed by SE_*T*_ = 10 lg(*P*_in_/*P*_out_) = SE_*A*_ + SE_*R*_ + SE_*I*_, where *P*_in_ and *P*_out_ are the power incident on and transmitted through a shielding material, respectively. The SE_*A*_ and SE_*R*_ are the absorption and reflection shielding efficiencies, respectively, and can be described as SE_*A*_ = 8.686 *αt* and SE_*R*_ = 20 lg |1 + *n*|^2^/4|*n*| [20]. For the composite with 30 wt.% CBC pyrolyzed at 1,200°C, using the measured electromagnetic parameters, we calculated the SE_*A*_ (with the thickness *t* assumed to be 2.0 mm) and the SE_*R*_, which together with SE_*A*_ + SE_*R*_ are shown in Figure 6b. It can be seen that the SE_*T*_ increased from 24 dB in the low frequencies to 39 dB at 18 GHz. The contribution to the SE_*T*_ was mainly from the reflection in the low frequency range and from the absorption in the high range. The EMI shielding efficiency is attributed to the formation of conducting interconnected nanofiber networks in an insulating paraffin wax matrix that will interact with the incident radiation and lead to the high shielding effectiveness.

## Conclusions

The pyrolysis of bacterial cellulose led to the formation of a unique interconnected web-like network of carbon nanoribbons, and this was used to fabricate carbon-matrix composites. These composites had remarkable imaginary permittivities and huge loss tangents and thus good attenuating properties. The web-like networks were very helpful for increasing the dielectric loss. The electromagnetic properties could be optimized by manipulating the bacterial nanoribbons by doping or surface modification; and thus, the RL and SE_*T*_ could be further improved. Based on these properties, and taking into account its other advantages, such as its light weight, easy processability, high mechanical strength, and good dispersion in the matrices, such CBC has the potential to be as an effective EMI shielding material and microwave absorber.

## Competing interests

The authors declare that they have no competing interests.

## Authors’ contributions

BD participated in the data analysis and wrote the manuscript. YR and YM participated in the detection of the SEM and TEM. GW, PZ, and SL participated in the design of the experiment and performed the data analysis. All authors read and approved the final manuscript.
